# A 3D Anisotropic Thermomechanical Model for Thermally Induced Woven-Fabric-Reinforced Shape Memory Polymer Composites

**DOI:** 10.3390/s23146455

**Published:** 2023-07-17

**Authors:** Yingyu Wang, Zhiyi Wang, Jia Ma, Chao Luo, Guangqiang Fang, Xiongqi Peng

**Affiliations:** 1 Institute of Aerospace System Engineering Shanghai, Shanghai 201108, China; petter_wong@163.com (Y.W.); 13611609242@139.com (Z.W.); ma_majia@163.com (J.M.); 2 Space Structure and Mechanism Technology Laboratory of China Aerospace Science and Technology Group Co., Ltd., Shanghai 201108, China; luochaoheu@163.com; 3 School of Materials Science and Engineering, Shanghai Jiao Tong University, Shanghai 200030, China; xqpeng@sjtu.edu.cn

**Keywords:** soft robotic gripper, shape memory polymer composites, woven fabric reinforcement, anisotropic, constitutive model

## Abstract

Soft robotic grippers offer great advantages over traditional rigid grippers with respect to grabbing objects with irregular or fragile shapes. Shape memory polymer composites are widely used as actuators and holding elements in soft robotic grippers owing to their finite strain, high specific strength, and high driving force. In this paper, a general 3D anisotropic thermomechanical model for woven fabric-reinforced shape memory polymer composites (SMPCs) is proposed based on Helmholtz free energy decomposition and the second law of thermodynamics. Furthermore, the rule of mixtures is modified to describe the stress distribution in the SMPCs, and stress concentration factors are introduced to account for the shearing interaction between the fabric and matrix and warp yarns and weft yarns. The developed model is implemented with a user material subroutine (UMAT) to simulate the shape memory behaivors of SMPCs. The good consistency between the simulation results and experimental validated the proposed model. Furthermore, a numerical investigation of the effects of yarn orientation on the shape memory behavior of the SMPC soft gripper was also performed.

## 1. Introduction

Soft robotic grippers can perform complex tasks in uncertain environments where it is difficult for rigid-bodied manipulators to perform tasks requiring flexibility. The main challenge faced by soft robotic grippers is that their stiffness can be variable during the grasping and transfer process [1]. During the grasping process, the stiffness of soft robotic grippers should be low to effectively buffer external impacts in complex environments and be able to adaptively grab irregularly shaped objects. During the object transfer process, the stiffness of soft robotic grippers should be high to maintain their configuration. Thermally induced shape memory polymer composites (SMPCs) are composed of thermally induced shape memory polymers (SMPs) and reinforcement. SMPs are a class of smart materials with the advantages of high stiffness, strength, and driving force, and SMPC-based soft actuators can be easily integrated with other adaptive functional components [2,3]. Furthermore, their stiffness varies with heat stimuli, which makes them a point of attraction in the field of soft robotic grippers [4,5,6,7]. 

The incorporation of a constitutive model is imperative to capture the thermomechanical and the shape memory behavior of SMPs and SMPCs. A great deal of research has been conducted on the thermomechanical modeling of SMPs. The modeling approaches of SMPs can be divided into two main types: the phase-transition-modeling approach and the viscoelastic modeling approach. The phase-transition-modeling approach assumes that SMPs are composed of a rubbery phase and a glass phase and that shape memory behavior can be realized by provoking the temperature induced transition between the glassy phase and the rubbery phase [8,9,10,11,12,13,14]. The viscoelastic modeling approach is based on the essential thermodynamic properties of SMPs and can be used to describe the entropy elasticity of SMPs above the glass transition temperature and the viscoelastic behavior below the glass transition temperature [15,16,17,18,19,20,21,22]. Moreover, there are also models that combine the concepts of phase transition and viscoelasticity theory [23,24,25,26,27].

Evidently, the constitutive models for SMPCs are more complicated than pure SMPs due to the introduction of reinforcements. Tan et al. [28] developed a constitutive model for unidirectional, continuous, carbon-fiber-reinforced SMPCs within a small strain range, and the effects of the inclination angle and the volume fraction of fiber on the thermomechanical and shape memory properties of SMPCs were investigated. Gu et al. [29] developed a finite strain viscoelastic model for unidirectional, continuous, fiber-reinforced SMPCs with internal state variables and considered the anisotropic thermal properties of SMPCs using a mesomechanics-based method. Li et al. [30] developed a thermomechanical model to describe the temperature-dependent elastic constants of unidirectional carbon-fiber-reinforced SMPCs with various fiber volume fractions based on the phase transition theory. Wang et al. [31] developed a constitutive model for unidirectional carbon-fiber-reinforced SMPCs that accounted for interfacial bonding strength. Hong et al. [32] developed a constitutive model based on energy decomposition that accounted for thermal residual stress. Su et al. [25] developed an anisotropic thermomechanical constitutive model for woven-fabric-reinforced SMPCs and investigated the effects of fiber yarn orientation on the shape memory properties of SMPCs. Although the above studies have made great contributions to the modelling of SMPCs, more efforts are needed to improve the accuracy of the corresponding models and facilitate their further development

In this paper, a novel 3D anisotropic thermomechanical model for thermally induced woven-fabric-reinforced SMPCs is developed based on Helmholtz free energy decomposition and the second law of thermodynamics. The total Helmholtz free energy is decomposed into the isotropic part of the matrix and the anisotropic part of the woven fabric. The energy of matrix part can be further decomposed into a hyperelastic part and a viscoelastic part, and the energy of the woven fabric part can be further decomposed into a hyperelastic part through the action of fiber stretching and fiber–fiber shearing. Furthermore, the rule of mixtures is modified to consider the stress distribution in the phases of the SMPCs, and stress concentration factors are introduced to consider fiber–matrix and fiber–fiber shearing interactions.

The paper is arranged as follows. Section 2 introduces a 3D anisotropic thermomechanical model for thermally induced woven-fabric-reinforced SMPCs. In Section 3, the model determination protocols for the material parameters’ and model’s verification are presented. Finally, the conclusions are drawn in Section 4.

## 2. Constitutive Model

### 2.1. Kinematics

**Figure 1:**
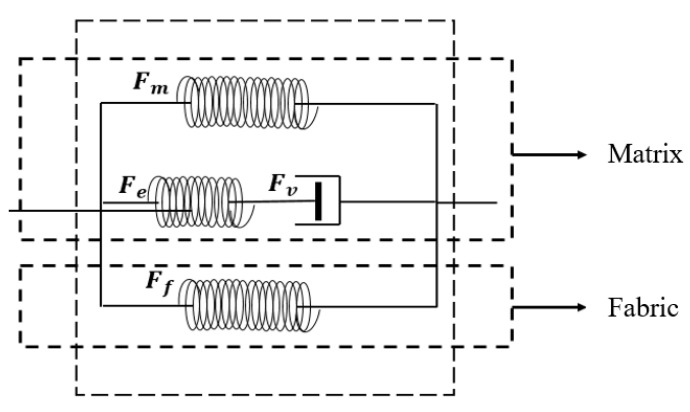
A rheological representation of the anisotropic thermomechanical model.

In this section, a 3D anisotropic thermomechanical model for thermally induced woven-fabric-reinforced SMPCs is proposed based on Helmholtz free energy decomposition and the second law of thermodynamics along with the consideration of the temperature-dependent interfacial effects of the SMPCs during the shape memory cycle. To keep matters simple, the anisotropic thermal deformation and residual stress of the SMPCs were not considered. The corresponding rheological model is shown in Figure 1. The subscripts *m*, *e*, *v*, and *f* denote the single spring element of the matrix part, the spring in the Maxwell element, the dashpot in the Maxwell element, and the single spring element of the fabric part, respectively.

In this case, perfect bonding between the fabric and matrix is assumed, which leads to the following relation:

(1) $$F_{f}=F_{m}=F$$

where F is the total deformation gradient.

F can be further decomposed into an elastic part $F_{e}$ and a viscous part $F_{v}$:

(2) $$F=F_{e}\cdot F_{v}$$

Then, the Cauchy–Green deformation tensors can be expressed as follows:

(3) $$\begin{array}{l} C=F_{}^{T}\cdot F_{},\;B=F_{}\cdot F_{}^{T}\\ C_{v}=F_{v}^{T}\cdot F_{v},\;B_{v}=F_{v}\cdot F_{v}^{T}\\ C_{e}=F_{e}^{T}\cdot F_{e}=(F_{}\cdot F_{v}^{-1}{)}^{T}\cdot F_{}\cdot F_{v}^{-1}=F_{v}^{-T}\cdot C\cdot F_{v}^{-1}\\ B_{e}=F_{e}\cdot F_{e}^{T}=F\cdot F_{v}^{-1}\cdot {\left(F\cdot F_{v}^{-1}\right)}^{T}=F_{}\cdot C_{v}{}^{-1}\cdot F_{}^{T} \end{array}$$

The Green–Lagrange tensors are denoted as follows:

(4) $$E=\frac{1}{2}\left(C-I\right)\;E_{e}=\frac{1}{2}\left(C_{e}-I\right)$$

where I is the second-order unit tensor.

The invariants of C and $C_{e}$ can be defined as follows:

(5) $$\begin{array}{l} I_{}^{1}=I_{m}^{1}=I_{f}^{1}tr\left(C\right),\;I_{}^{2}=I_{m}^{2}=I_{f}^{2}\frac{1}{2}\left[{\left(trC\right)}^{2}-tr{\left(C\right)}^{2}\right],\;I_{}^{3}=I_{m}^{3}=I_{f}^{3}\det\left(C\right)\;\\ I_{e}^{1}=tr\left(C_{e}\right),\;I_{e}^{2}=\frac{1}{2}\left[{\left(trC_{e}\right)}^{2}-tr{\left(C_{e}\right)}^{2}\right],\;I_{e}^{3}=\det\left(C_{e}\right)\; \end{array}$$

**Figure 2:**
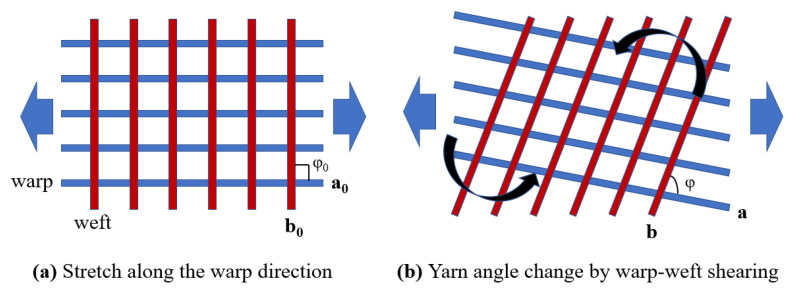
The yarn orientation unit vectors.

The yarn orientation unit vectors in the original configuration are denoted as $a_{0}$ and $b_{0}$, as shown in Figure 2a, and the yarn orientation unit vectors in the current configuration are denoted as a, b, which can be formulated as follows:

(6) $$a=\frac{a_{0}\cdot F_{}\;}{\left\|a_{0}\right\|}\;b=\frac{b_{0}\cdot F_{}\;}{\left\|b_{0}\right\|}$$

The main modes of the deformation of the fabrics mainly include tension along the yarn orientation and shearing between the weft and warp yarn. To analyze the mechanical behavior of the fabrics, the invariants $I_{4}^{}$ and $I_{7}^{}$ are introduced [33]:

(7) $$\begin{array}{l} I_{a}^{4}=a_{0}\cdot C\cdot a_{0},\;I_{b}^{4}=b_{0}\cdot C\cdot b_{0}\\ I_{}^{7}=a_{0}\cdot C^{2}\cdot b_{0} \end{array}$$

### 2.2. Constitutive Model for Matrix SMPs

It can be seen from Figure 1 that the total Helmholtz free energy of SMPs $\psi_{matrix}$ can be decomposed into $\psi_{m}$ and $\psi_{e}$:

(8) $$\psi_{matrix}\left(C,C_{e},T\right)=\psi_{m}\left(C,T\right)+\psi_{e}\left(C_{e},T\right)$$

Based on Equation (8), ${\dot{\psi }}_{matrix}$ can be formulated as

(9) $${\dot{\Psi }}_{matrix}=\frac{\partial \Psi_{m}}{\partial C}:\dot{C}+\frac{\partial \Psi_{e}}{\partial C_{e}}:{\dot{C}}_{e}+\frac{\partial \Psi_{m}}{\partial T}\dot{T}+\frac{\partial \Psi_{e}}{\partial T}\dot{T}$$

The Clausius–Duhem inequality based on the second law of thermodynamics can be expressed as follows [10]:

(10) $$S:\dot{E}-\left(\dot{\Psi }+\dot{T}\eta \right)-q\cdot \nabla T/T\geq 0$$

where S is the second Piola–Kirchhoff stress tensor, η is entropy, q is the heat flux vector, and ∇ represents the gradient operator.

Substituting Equations (4) and (9) into (10) leads to

(11) $$\begin{array}{l} \frac{1}{2}\left[S-2\frac{\partial \psi_{m}}{\partial C}-2F_{v}^{-1}\frac{\partial \psi_{e}}{\partial C_{e}}F_{v}^{-T}\right]:\dot{C}-2C_{e}\cdot \frac{\partial \psi_{e}}{\partial C_{e}}:L_{v}-\left(\frac{\partial \psi_{m}}{\partial T}+\frac{\partial \psi_{e}}{\partial T}+\eta \right)\dot{T}\\ -q\cdot \nabla T/T\geq 0 \end{array}$$

where $L_{v}={\dot{F}}_{v}\cdot F_{v}{}^{-1}$ is the velocity gradient tensor.

Equation (11) must account for arbitrary thermodynamic processes. Therefore, the second Piola–Kirchhoff stress of matrix $S_{matrix}$ and the velocity gradient tensor of the viscoelastic part can be expressed as follows:

(12) $$\begin{array}{l} S_{matrix}=2\frac{\partial \psi_{m}}{\partial C}+2F_{v}^{-1}\cdot \frac{\partial \psi_{e}}{\partial C_{e}}\cdot F_{v}^{-T}\\ L_{v}=\frac{1}{\zeta }C_{e}\cdot \frac{\partial W_{e}}{\partial C_{e}} \end{array}$$

where ζ is a temperature-dependent viscosity parameter, which can be expressed as follows:

(13) $$\zeta \left(T\right)=\phi \zeta_{\_l}+\zeta_{\_h}\exp\left(\frac{A}{T}\right)\;$$

where subscript *l* denotes parameters at $T_{l}$, which is the lowest temperature below the glass transition temperature ($T_{g}$) in a thermomechanical test. Subscript *h* denotes the parameters at $T_{h}$, which is the highest temperature above $T_{g}$ in a thermomechanical test. A is a material parameter, while ϕ is a weight function, which can be expressed as follows:

(14) $$\phi =\frac{1}{1+\exp\left[g\left(T-T_{r}\right)\right]}$$

where g is a material parameter, and $T_{r}$ is the reference temperature.

Here, the Mooney–Rivlin model is adopted for $\psi_{m}$ and $\psi_{e}$:

(15) $$\begin{array}{l} \psi_{m}=C_{m}^{10}\left[{\left(I_{m}^{3}\right)}^{-\frac{1}{3}}I_{m}^{1}-3\right]+C_{m}^{01}\left[{\left(I_{m}^{3}\right)}^{-\frac{2}{3}}I_{m}^{2}-3\right]+\frac{1}{D_{m}}{\left[{\left(I_{m}^{3}\right)}^{\frac{1}{2}}-1\right]}^{2}\\ \psi_{e}=C_{e}^{10}\left[{\left(I_{e}^{3}\right)}^{-\frac{1}{3}}I_{e}^{1}-3\right]+C_{e}^{01}\left[{\left(I_{e}^{3}\right)}^{-\frac{2}{3}}I_{e}^{2}-3\right]+\frac{1}{D_{e}}{\left[{\left(I_{e}^{3}\right)}^{\frac{1}{2}}-1\right]}^{2} \end{array}$$

where $C_{m}^{10}$, $C_{e}^{10}$, $C_{m}^{01}$, $C_{e}^{01}$, $D_{m}$, and $D_{e}$ are temperature-dependent parameters, which can be expressed as follows:

(16) $$P\left(T\right)=\phi P_{\_l}+P_{\_l}\exp\left(\frac{A}{T}\right),\;\left(P=C_{m}^{10},C_{e}^{10},C_{m}^{01},C_{e}^{01},D_{m},D_{e}\right)$$

Here, the isochoric flow assumption ($\left|F_{v}\right|=1$) leads to the following relation:

(17) $$I_{m}^{3}=I_{e}^{3}\;D_{m}=D_{e}=D$$

Based on Equations (12)–(17), the Cauchy stress of the matrix SMPs and the velocity gradient of the viscoelastic part can be expressed as follows:

(18) $$\begin{array}{l} \sigma_{matrix}={\left(I_{m}^{3}\right)}^{-\frac{1}{2}}F\cdot S_{matrix}\cdot F^{T}\\ \;=2C_{m}^{10}{\left(I_{m}^{3}\right)}^{-\frac{5}{6}}\left(B_{m}-\frac{1}{3}I_{m}^{1}I\right)+2C_{m}^{01}{\left(I_{m}^{3}\right)}^{-\frac{7}{6}}\left[\left(I_{m}^{1}I-C_{m}\right)B_{m}-\frac{2}{3}I_{m}^{2}I\right]\\ \;+2C_{e}^{10}{\left(I_{m}^{3}\right)}^{-\frac{1}{2}}{\left(I_{e}^{3}\right)}^{-\frac{1}{3}}\left(B_{e}-\frac{1}{3}I_{e}^{1}I\right)+2C_{e}^{01}{\left(I_{m}^{3}\right)}^{-\frac{1}{2}}{\left(I_{e}^{3}\right)}^{-\frac{2}{3}}\\ \;\left[\left(I_{e}^{1}I-C_{e}\right)B_{e}-\frac{2}{3}I_{e}^{2}I\right]+\frac{2}{D}\left({\left(I_{m}^{3}\right)}^{\frac{1}{2}}-1\right)I\\ L_{v}=\frac{1}{\zeta }\left\{C_{e}^{10}{\left(I_{e}^{3}\right)}^{-\frac{1}{3}}\left(C_{e}-\frac{1}{3}I_{e}^{2}I\right)+C_{e}^{01}{\left(I_{e}^{3}\right)}^{-\frac{2}{3}}\left[\left(I_{e}^{1}I-C_{e}\right)C_{e}-\frac{2}{3}I_{e}^{2}I\right]\right.\\ \;+\left.\frac{1}{D}{\left(I_{e}^{3}\right)}^{\frac{1}{2}}\left[{\left(I_{e}^{3}\right)}^{\frac{1}{2}}-1\right]I\right\} \end{array}$$

### 2.3. Constitutive Model for Fabric

The woven fabric yarn can be stretched along the fiber orientation and sheared at a wider angle when a load is applied. Therefore, the total Helmholtz free energy of the fabric $\psi_{fabric}$ can be decomposed into a fiber-stretching part $\psi_{ten}$ and a fiber-shearing part $\psi_{shr}$:

(19) $$\Psi_{fabric}\left(I_{a}^{4},I_{b}^{4},I_{}^{7}\right)=\Psi_{ten}\left(I_{a}^{4},I_{b}^{4}\right)+\Psi_{shr}\left(I_{}^{7}\right)$$

Here, the polynomial function of $I_{a}^{4},I_{b}^{4}$ was adopted for $\psi_{ten}$, and $I_{a}^{4},I_{b}^{4}$ are assumed to be equal to 1 under the following condition [33]:

(20) $$\begin{array}{l} \Psi_{ten}\left(I_{a}^{4},I_{b}^{4}\right)=k_{1}\left[{\left(I_{a}^{4}-1\right)}^{4}+{\left(I_{b}^{4}-1\right)}^{4}\right]+k_{2}\left[{\left(I_{a}^{4}-1\right)}^{3}+{\left(I_{b}^{4}-1\right)}^{3}\right]\\ \;+k_{3}\left[{\left(I_{a}^{4}-1\right)}^{2}+{\left(I_{b}^{4}-1\right)}^{2}\right] \end{array}$$

where $k_{1},k_{2},k_{3}$ are material parameters.

Here, the polynomial function of $I_{}^{7}$ was adopted for $\psi_{shr}$ [33]:

(21) $$\Psi_{shr}\left(I_{}^{7}\right)=k_{4}{\left(I_{}^{7}\right)}^{4}+k_{5}{\left(I_{}^{7}\right)}^{3}+k_{6}{\left(I_{}^{7}\right)}^{2}$$

where $k_{4},k_{5},k_{6}$ are material parameters.

By substituting Equations (19)–(21) into Equation (10), the Cauchy stress of fabric can be expressed as follows:

(22) $$\begin{array}{l} \sigma_{fabric}={\left(I_{}^{3}\right)}^{-\frac{1}{2}}F\cdot S_{fabric}\cdot F^{T}\\ \;={\left(I_{}^{3}\right)}^{-\frac{1}{2}}\left[2I_{a}^{4}\frac{\partial \Psi_{ten}}{\partial I_{a}^{4}}-\left(I_{}^{7}+a_{0}\cdot b_{0}\right)\frac{\partial \Psi_{shr}}{\partial I_{}^{7}}\right]a⊗a\\ \;+{\left(I_{}^{3}\right)}^{-\frac{1}{2}}\left[2I_{b}^{4}\frac{\partial \Psi_{ten}}{\partial I_{4}^{b}}-\left(I_{}^{7}+a_{0}\cdot b_{0}\right)\frac{\partial \Psi_{shr}}{\partial I_{}^{7}}\right]b⊗b\\ \;+{\left(I_{}^{3}\right)}^{-\frac{1}{2}}\frac{\partial \Psi_{shr}}{\partial I_{}^{7}}\left(a⊗b+b⊗a\right) \end{array}$$

Generally, the total stress of SMPCs σ can be formulated using the rule of mixtures:

(23) $$\sigma =v_{m}\sigma_{matrix}+v_{f}\sigma_{fabric}$$

where $v_{m},v_{f}$ are the volume fractions of the matrix SMPs and fabric, respectively, and $v_{m}+v_{f}=1$.

However, as demonstrated in previous experiments [25,31,34], the total stress of SMPCs cannot be accurately described by the rule of mixtures alone since a phase transition of the SMPs will occur with a temperature change, and the stress distribution in the phases of the SMPCs are also related to their microstructure. Therefore, an effective temperature-dependent fabric volume fraction of fabric is introduced based on phase transition theory, and Equation (23) is modified as follows:

(24) $$\begin{array}{l} \sigma =(1-v_{f})\sigma_{matrix}+\gamma_{inter}v_{f}\sigma_{fabric}\\ v_{f}=\frac{v_{f\_ref}}{1+\exp\left[g\left(T-T_{r}\right)\right]} \end{array}$$

where $v_{f\_ref}$ denotes the reference volume fractions of the fabric, and $\gamma_{inter}$ denotes the stress concentration factors to consider fiber–fiber and matrix-fiber shearing interactions, which are assumed to be equal to 1 when stretching along the yarn.

## 3. Parameters for Determining Protocol and Model Verification

### 3.1. Methods for Determining Matrix SMPs Material Parameters

The material parameters in the matrix SMP part can be determined following the protocol outlined in our previous works [26].

### 3.2. Methods for Determining Remaining Material Parameters

With the parameters of the matrix SMP part determined, the parameters of $k_{1},\;k_{2},\;k_{3}$ in $\psi_{ten}$ and $k_{4},k_{5},k_{6}$ in $\psi_{shr}$ and $v_{f\_ref}$ can be determined using the following procedure:

1. Based on the Equations (18) and (22)–(24), $k_{1},\;k_{2},\;k_{3}$ can be determined by fitting the stress–strain curve of the 0° SMPCs at $T_{l}$ with an initial assumption of $v_{f\_ref}=v_{f}$.
2. With $k_{1},\;k_{2},\;k_{3}$ determined, $k_{4},k_{5},k_{6}$ can be obtained by fitting the stress–strain curve of the bias tension of the SMPCs based on Equations (18) and (22)–(24).
3. $v_{f\_ref}$ can be obtained by fitting the curve of the strain of the 0° SMPCs in the loading step, cooling step, and unloading step in the shape memory cycle.
4. Compare the fitting results with the experimental data. If good consistency has been achieved, then these parameters are determined. If not, modify the initial estimate of the constant $v_{f\_ref}$ and return to (1).
5. With the above parameter determined, $\gamma_{inter}$ can be obtained by fitting the curve of the strain of the 45° SMPCs in the loading step, cooling step, and unloading step in the shape memory cycle.

**Figure 3:**
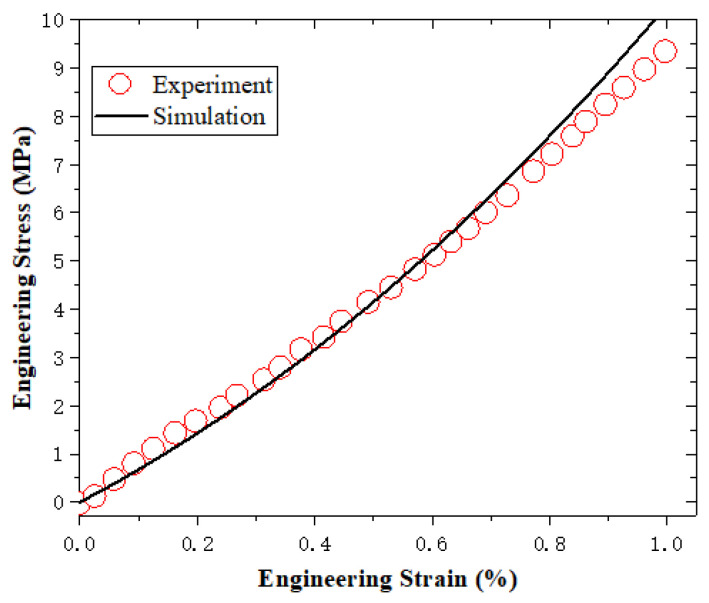
The fitting results regarding $k_{1},\;k_{2},\;k_{3}$.

**Figure 4:**
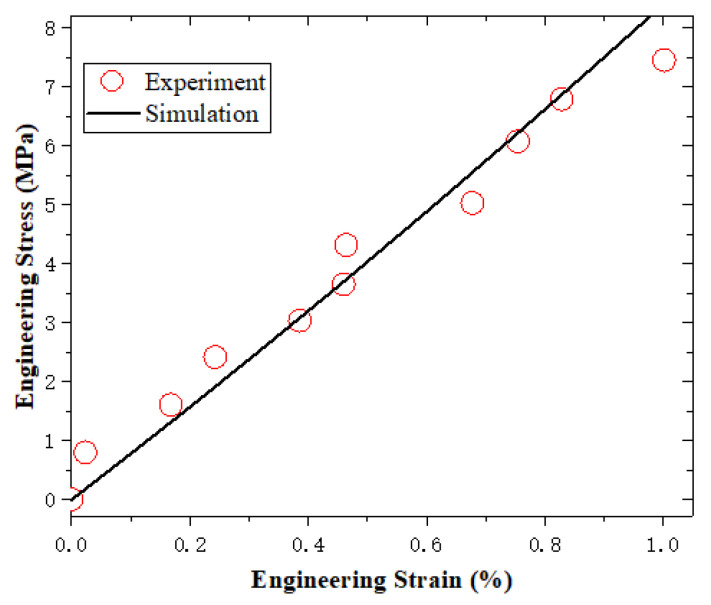
The fitting results regarding $k_{4},k_{5},k_{6}$.

**Figure 5:**
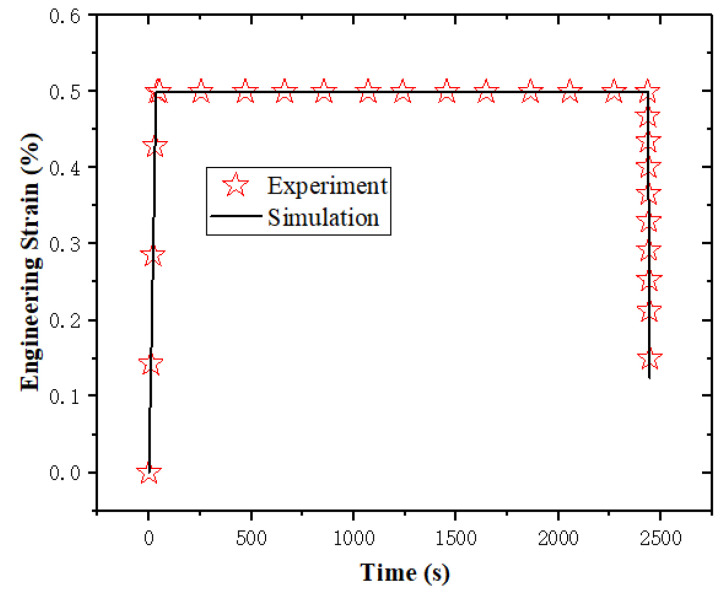
The fitting results regarding $v_{f\_ref}$.

**Figure 6:**
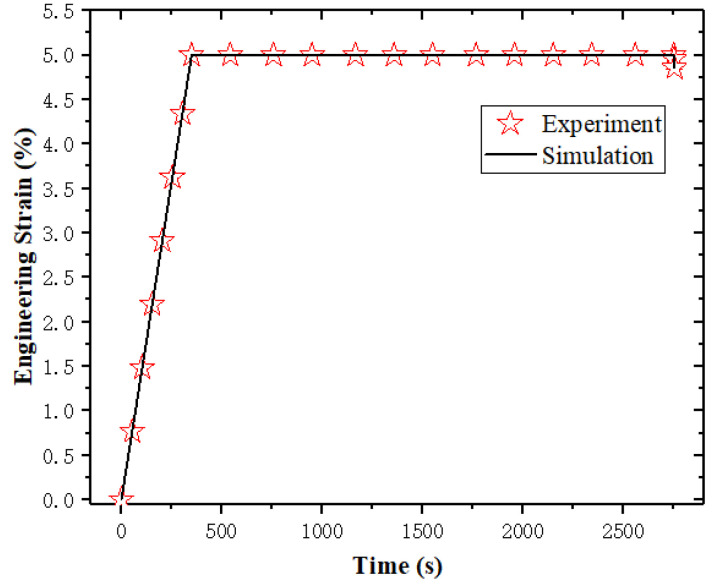
The fitting results regarding $\gamma_{inter}$.

According to the above parameter determination protocol, the parameters of $k_{1},\;k_{2},\;k_{3}$ in $\psi_{ten}$ and $k_{4},k_{5},k_{6}$ in $\psi_{shr}$ and $v_{f\_ref}$ are shown). All determined material parameters are summarized in Table 1, and the fitting results regarding *k*_1_, *k*_2_, *k*_3_, *k*_4_, *k*_5_, *k*_6_, $v_{f\_ref}$, and $\gamma_{inter}$ are shown in Figure 3, Figure 4, Figure 5 and Figure 6.

(25) $$\begin{array}{l} k_{1}=3690\left(\mathrm{MPa}\right),\;k_{2}=3650\;\left(\mathrm{MPa}\right),\;k_{3}=115\left(\mathrm{MPa}\right)\;\\ k_{4}=300\left(\mathrm{MPa}\right),\;k_{5}=220\;\left(\mathrm{MPa}\right),\;k_{6}=170\left(\mathrm{MPa}\right)\;\\ v_{f\_ref}=0.7,\;\gamma_{inter}=0.01 \end{array}$$

### 3.3. Model Verification

The proposed model was implemented in the commercial finite element software package ABAQUS/Standard via a user material subroutine (UMAT) to simulate the shape memory tests carried out in the study by Su et al. [25]. Eight-node linear hexahedron continuum elements (C3D8) are used. Loading and boundary conditions are set according to the design of the experiment. Heat transfer is ignored here, and the temperature is applied through the predefined field. 

**Figure 7:**
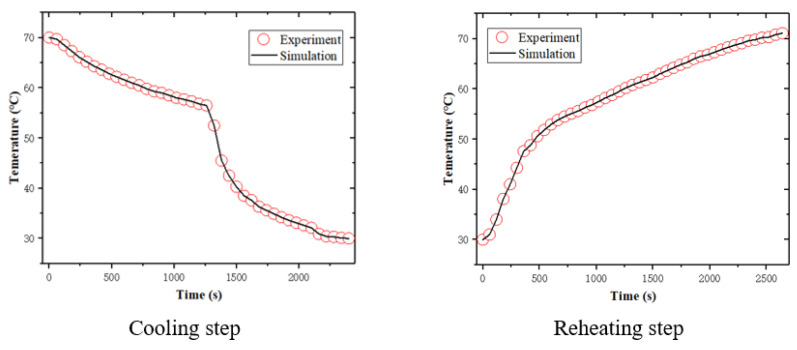
The temperature histories in the cooling and reheating steps.

**Figure 8:**
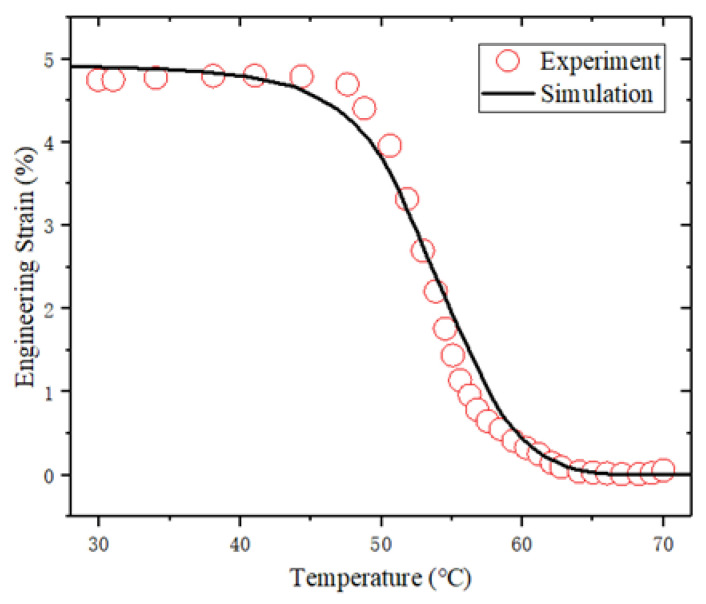
Comparison between experiment and simulation results regarding shape recovery for SMPs.

First, the shape memory tests of the matrix SMPs were simulated. The temperature histories of the simulation in the cooling and reheating steps are consistent with the experimental data, as shown in Figure 7. During the shape memory cycle tests, the temperature of the experimental specimens cannot immediately reach the temperature of the experimental equipment because it takes time for the specimens to reach thermal equilibrium. Therefore, a temperature lag of 3.7 °C for SMP was assumed. The simulation results regarding the matrix SMPs are shown in Figure 8. It can be seen that the simulation results are in good agreement with the experimental data, thus verifying the effectiveness of the proposed model in predicting the shape memory behavior of matrix SMPs.

**Figure 9:**
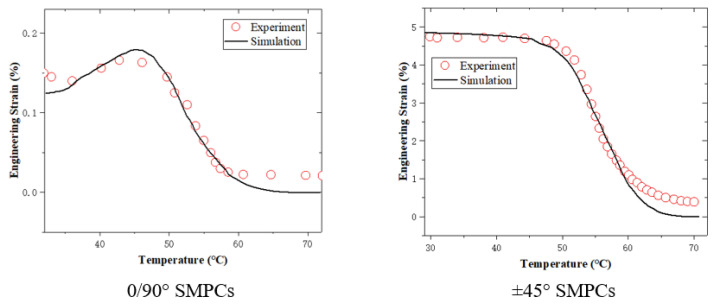
Comparison between experimental and simulation results regarding shape recovery for SMPCs.

Second, the shape memory tests of the SMPCs with an initial yarn orientation of 0/90° and ±45° were simulated. The temperature histories of the simulation in the cooling and reheating steps are consistent with the experimental data, as shown in Figure 7. Temperature lag values of 3.2 °C and 3.7 °C for the 0/90° and ±45° woven-fabric-reinforced SMPCs were assumed. The simulation results are shown in Figure 9. It can be seen that the simulation accurately reproduces the shape memory recovery behavior of the SMPCs, which demonstrates the validity of the proposed model in predicting the shape memory behavior of SMPCs. The deviation between the simulation results and the experimental data in the final recovery stage might have been caused by interfacial failure and internal stress, and this deviation will be modified by introducing anisotropic thermal and stress internal stress and interfacial failure in our future research.

**Figure 10:**
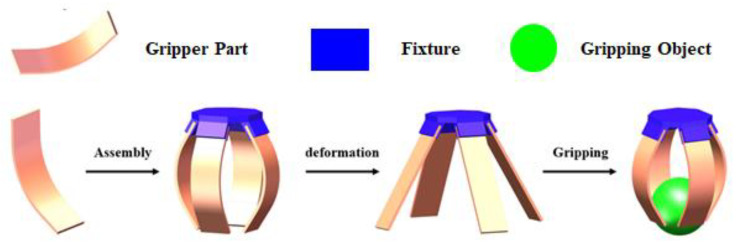
The soft robotic gripper’s work process.

**Figure 11:**
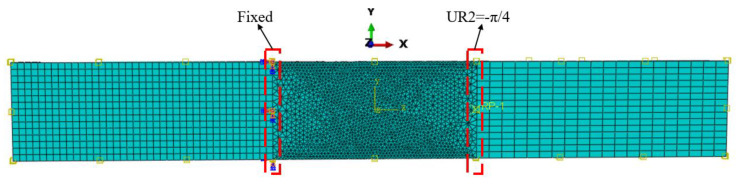
The finite element model of individual SMPC’s soft gripper part.

Finally, the shape memory behavior of the SMPC gripper part was simulated to investigate the effects of yarn orientation. The main deformation mode of a soft robotic gripper is flexural deformation, as shown in Figure 10, since the human hand’s ability to grasp objects is achieved through the movement of bones and joints. Here, the grasping of objects via hands is simulated through the flexural deformation of the individual gripper part with dimensions of 70 mm × 25 mm × 2 mm. Eight-node linear hexahedron continuum elements (C3D8) and six-node linear triangular prism elements (C3D6) are used. The applied position of the load and the boundary conditions are shown in Figure 11. The left area of the fixed boundary and the right area of the displacement boundary represent the two finger bones, and the middle part represents the flexible joint. 

**Figure 12:**
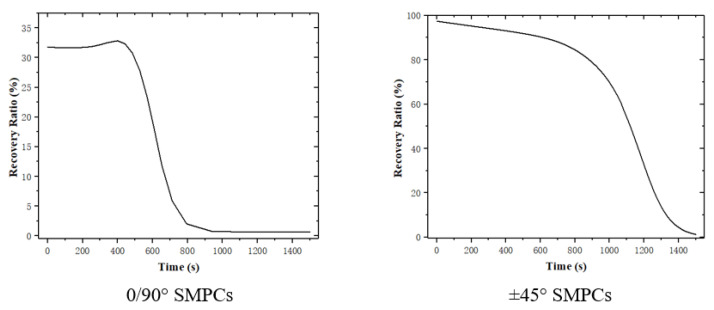
The shape recovery of individual SMPC’s soft gripper part.

**Figure 13:**
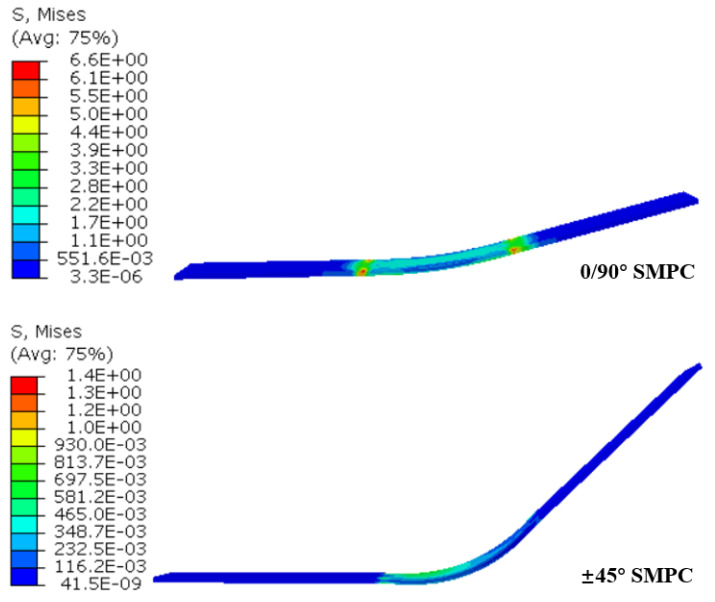
The stored stress of individual SMPC’s soft gripper part at the beginning of the reheating step.

Initially, the temperature was set at 70 °C, and a rotation boundary condition of UR2 =−π/4 was applied. Then, the temperature was set to decrease from 70 °C to 20 °C at a cooling rate of −2 °C/min, which allowed for the shape to be maintained, followed by unloading when the temperature reached 20 °C. Finally, the temperature was set to increase from 20 °C to 70 °C at a heating rate of 2 °C/min in the free state. The simulation results are shown in Figure 12 and Figure 13.

**Figure 14:**
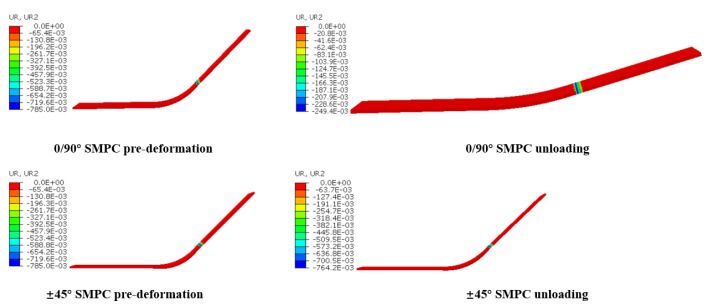
The change in the angle radian values of the 0/90° and ±45° SMPCs in the pre-deformed stage and the unloading step.

It can be seen from Figure 12 that the recovery rate of the 0/90° SMPCs is faster than that of the ±45°SMPCs owing to their larger stored recovery stress, as shown in Figure 13. However, the shape fixity ratio of the 0/90° SMPCs is lower than that of the ±45° SMPCs, as shown in Figure 14, because the fabric used in this paper was carbon fabric; additionally, as the fiber orientation angle decreased, the reinforcing effect of the carbon fiber was enhanced; thus, the carbon fiber was rendered elastic. When SMPCs are stretched at a high temperature and unloaded after cooling, the larger rebound stress of the fiber reduces the shape fixation rate of the 0/90 SMPCs compared to the ±45 SMPCs. Therefore, it is necessary to comprehensively consider the response rate and accuracy of an SMPC soft gripper to design an appropriate fiber orientation in practical applications.

## 4. Conclusions

An anisotropic thermomechanical model for thermally induced woven-fabric-reinforced SMPCs was developed based on Helmholtz free energy decomposition and the second law of thermodynamics. The total Helmholtz free energy of the SMPCs was decomposed into an isotropic visco-hyperelastic matrix part and an anisotropic hyperelastic fabric part. The stress distribution of the phases in the SMPCs was described using a modified rule of mixtures based on phase transition theory, and stress concentration factors were introduced to consider fiber–matrix and fiber–fiber shearing interactions. The shape memory tests presented in the study by Su et al. [25] were simulated, and a comparison between the simulation results and the experimental data verified the effectiveness of the proposed model. Finally, the effects of yarn orientation on the shape memory behavior of the soft robotic gripper were investigated using the proposed model.

## Figures and Tables

**Table 1:** Material parameters of the proposed model.

	Parameters	Value	Unit
Matrix	$C_{m}^{10},C_{m}^{01},C_{e}^{10}{}_{\_l},C_{e}^{01}{}_{\_l}$	1.8, 0.9, 160, 25	MPa
	A	−2850	K^−1^
	g	0.25	°C^−1^
	$T_{r}^{}$	43	°C
	$D_{\_l}$	5.57 × 10^−3^	MPa^−1^
	$\xi_{\_l}$	5.0 × 10^4^	MPa·s
Fiber	k1,k2,k3	3690, 3620, 115	MPa
	k4,k5,k6	300, 220, 170	MPa
	$v_{f\_ref},\gamma_{inter}$	0.7, 0.01	
